# G protein-coupled receptor kinase 2 regulates mitochondrial bioenergetics and impairs myostatin-mediated autophagy in muscle cells

**DOI:** 10.1152/ajpcell.00516.2018

**Published:** 2019-07-03

**Authors:** Leandro Henrique Manfredi, Joshur Ang, Nesibe Peker, Ruben K. Dagda, Craig McFarlane

**Affiliations:** ^1^Department of Physiology, Medical School of Ribeirão Preto, University of São Paulo, Ribeirão Preto, Brazil; ^2^Federal University of Fronteira Sul, Medical School, Chapecó, Santa Catarina, Brazil; ^3^Singapore Institute for Clinical Sciences, Agency for Science, Technology, and Research (A*STAR), Brenner Centre for Molecular Medicine, Singapore; ^4^School of Biological Sciences, Nanyang Technological University, Singapore; ^5^Department of Pharmacology, School of Medicine, University of Nevada, Reno, Nevada; ^6^Department of Molecular and Cell Biology, College of Public Health, Medical, and Veterinary Sciences, James Cook University, Townsville, Queensland, Australia

**Keywords:** autophagy, GRK2, mitochondria, myoblast, myostatin

## Abstract

G protein-coupled receptor kinase 2 (GRK2) is an important protein involved in β-adrenergic receptor desensitization. In addition, studies have shown GRK2 can modulate different metabolic processes in the cell. For instance, GRK2 has been recently shown to promote mitochondrial biogenesis and increase ATP production. However, the role of GRK2 in skeletal muscle and the signaling mechanisms that regulate GRK2 remain poorly understood. Myostatin is a well-known myokine that has been shown to impair mitochondria function. Here, we have assessed the role of myostatin in regulating GRK2 and the subsequent downstream effect of myostatin regulation of GRK2 on mitochondrial respiration in skeletal muscle. Myostatin treatment promoted the loss of GRK2 protein in myoblasts and myotubes in a time- and dose-dependent manner, which we suggest was through enhanced ubiquitin-mediated protein loss, as treatment with proteasome inhibitors partially rescued myostatin-mediated loss of GRK2 protein. To evaluate the effects of GRK2 on mitochondrial respiration, we generated stable myoblast lines that overexpress GRK2. Stable overexpression of GRK2 resulted in increased mitochondrial content and enhanced mitochondrial/oxidative respiration. Interestingly, although overexpression of GRK2 was unable to prevent myostatin-mediated impairment of mitochondrial respiratory function, elevated levels of GRK2 blocked the increased autophagic flux observed following treatment with myostatin. Overall, our data suggest a novel role for GRK2 in regulating mitochondria mass and mitochondrial respiration in skeletal muscle.

## INTRODUCTION

G protein-coupled receptor kinases (GRKs) are serine/threonine kinases initially identified to participate in the process of G protein-coupled receptor desensitization (53). GRKs comprise a family that can be partitioned into three groups through sequence homology: GRK1/7, GRK2/3, and GRK4/5/6 (54). GRK1 and 7 are found in retinal rods and cones, respectively, and GRK4 is expressed in testis, cerebellum, and kidney (38, 53–55). However, ubiquitous expression of GRK2, 3, 5, and 6 is observed in mammalian tissues (53–55). These kinases can phosphorylate specific amino acid residues in the intracellular domain of activated receptors and lead to recruitment of adaptor proteins (e.g., β-arrestins) to attenuate intracellular G-protein signaling (54, 63).

Recent studies have identified GRK2 as an emerging kinase involved in regulating different cellular process through phosphorylation and/or association with other proteins (13, 23, 26, 72). Moreover, GRK2 expression and activity are tightly regulated and are altered during several pathological conditions, for example, hypertension, heart failure, and inflammation (41, 49, 75). GRK2 has recently been linked to mitochondrial function and biogenesis (18). Overexpression of GRK2 has been shown to promote increased mitochondrial mass and further enhance ATP production due to the ability of GRK2 to target and phosphorylate mitochondrial proteins in human embryonic kidney 293 (HEK293) cells, whereas knockdown of GRK2 led to reduced ATP production in skeletal muscle (18). Moreover, macrophages treated with LPS exhibited enhanced GRK2 accumulation in mitochondria, which was associated with increased mtDNA copy and reduced reactive oxygen species production (65). Although several studies have helped to delineate GRK2 function using different model systems, the function of GRK2 in skeletal muscle metabolism remains to be fully elucidated.

Members of the transforming growth factor-β superfamily of secreted growth factors, including GDF11 and myostatin, have negative impact on skeletal muscle growth and maintenance (11, 45). More specifically, myostatin has been previously shown to inhibit myoblast proliferation (58, 69) and myogenic differentiation (27, 33), block protein synthesis signaling, and promote a reduction in myotube size (70). Moreover, elevated levels myostatin has been shown to promote loss of mitochondrial membrane potential and impair mitochondrial function in cancer cells (39). Importantly, myostatin is a potent inducer of skeletal muscle wasting and increased myostatin activity has been observed in different atrophic conditions (1, 42, 56).

Over the past 10 years, many studies have revealed the pathological mechanisms involved in myostatin-mediated atrophy in skeletal muscle. Specifically, McFarlane et al. (44) reported that myostatin was able to block insulin-like growth factor-1 (IGF-1)/phosphatidyinositol 3 phosphate kinase (PI3K)/Akt signaling and activate the transcription factor forkhead box O1 (FoxO1), which increases the expression of muscle atrophy F-box (MAFbx)/atrogin-1 and muscle ringer finger 1 (MuRF1)/Trim63, two well-known muscle-specific E3 ligases that are associated with muscle atrophy (2, 12). Myotubular atrophy has also been noted in human myotubes upon treatment with excess myostatin (32, 40), which was associated with increased levels of atrogin-1 and MuRF1 (40). Additional work has revealed that myostatin signals through Smad3 to increase FoxO1 and atrogin-1 to promote the ubiquitination and subsequent loss of critical sarcomeric proteins, such as myosin heavy chain (MyHC), during muscle wasting (40).

As GRK2 and myostatin have been shown to regulate mitochondrial function, we sought to determine a potential role for myostatin in regulating GRK2 and subsequent mitochondrial respiration in skeletal muscle. In this report, we show that myostatin targets and suppresses GRK2 protein levels in muscle cells, through a mechanism involving the ubiquitin-proteasome pathway (UPP). In the present study, we find that myostatin treatment leads to impaired mitochondrial respiration, which was associated with mitochondrial fragmentation, enhanced autophagic flux, and reduced mitochondrial content in muscle cells. We have further unraveled a novel role for GRK2 in regulating mitochondrial respiration in muscle cells. Overexpression of GRK2 in myoblasts also led to increased mitochondrial fragmentation; however, unlike myostatin, GRK2 overexpression was associated with enhanced mitochondrial respiration and increased mitochondrial mass. Surprisingly, while overexpression of GRK2 was not able to overcome the negative effect of excess myostatin on mitochondria respiration; elevated GRK2 levels resulted in increased mitochondria content and a reduction in the overt autophagic flux noted in the presence of excess myostatin. Overall, these data reveal a novel role for GRK2 in regulating mitochondrial respiration and mass in muscle cells and reveal that increased expression of GRK2 may act to compensate, at least in part, for the loss of mitochondria noted upon myostatin treatment.

## MATERIALS AND METHODS

### 

#### Cell culture and treatments.

Mouse C2C12 myoblasts were obtained from American Type Culture Collection and their maintenance has been previously described (43). C2C12 myoblasts were expanded in myoblast proliferation medium (10% FBS, 1% P/S, and DMEM; Invitrogen, Carlsbad, CA) and differentiated into myotubes through serum withdrawal in differentiation medium (DMEM, 2% HS, and 1% P/S; Invitrogen) for 96 h to ensure complete differentiation of cultures. Doxycycline (2 µg/mL) was added together with differentiation medium to induce the stable overexpression of GRK2. Recombinant myostatin protein (Mstn) was purified from *Escherichia coli* (62) and was used at a concentration of 3 μg/mL for cell treatments, unless otherwise stated. For proteasome inhibitor studies, C2C12 myotubes were treated with 3 μg/mL recombinant Mstn for a total period of 24 h. To block the activity of the proteasome, MG132 (Sigma, St. Louis, MO) and epoxomicin (Epox; Sigma) chemicals were added to C2C12 myotubes at 10 μM and 100 nM final concentrations, respectively, 10 h prior to harvesting the cells. The difference in total GRK2 seen in the absence of presence of the proteasome inhibitors represents the content of GRK2 that is being degraded through the ubiquitin-proteasome system (29, 34). One independent experiment was performed with MG132 with three biological replicates, and one confirmatory experiment was performed with Epox.

To block the lysosomal pathway, 100 µM of chloroquine (Sigma) were added to myotubes in the presence or absence of Mstn (3 μg/mL) for 12 h. The difference in the protein levels of LC3-II between samples treated with and without chloroquine represents the level of autophagic flux in the cells (30, 77). Two different experiments were performed, each with one biological replicate.

#### Generation of GRK2 stable cell lines.

Full-length murine *Grk2* cDNA (NM_130863.2) was PCR amplified using the following primers: 5′-CC ACC GGT ATG CAG AAG TAT CTG GAG GAC CGA-3′ and 5′-ACC TGT ACA TCA GAG GCC GTT GGC ACT GCC ACG-3′ and cloned into the pGEM-T easy cloning vector (Promega). After sequence verification, *Grk2* was subcloned into the doxycycline-inducible PEM777 expression vector (28). *Grk2*-PEM777 or empty-PEM777 (control) was transfected into C2C12 myoblasts and cells using Lipofectamine 2000 (Invitrogen), as previously described (28). Following 3 days of selection with puromycin (1 µg/mL), stably transfected cells were harvested and expanded for further experimental procedures in the presence of 2 µg/mL doxycycline.

#### Assessment of mitochondrial respiration.

Mitochondrial respiration was assessed in vitro using the XF^e^24 extracellular flux analyzer and the XF Cell Mito Stress and Glycolysis Stress Test Kits, as per the manufacturer’s protocol (Agilent Technologies, Santa Clara, CA) and as described previously (20, 52). For assessment of real-time mitochondrial respiration of myotubes, myoblasts were seeded (10,000 cells/well) onto XF^e^24 cell culture microplates and differentiated to form myotubes, as outlined above, in the presence of 100 ng/mL doxycycline for 48 h. Cells were then treated with either 2 µg/mL recombinant Mstn protein or an equal volume of dialysis buffer (control) for a further 24 h. The XF^e^24 sensor cartridge was hydrated overnight at 37°C in a non-CO_2_ incubator. Thirty minutes before assay run, differentiation medium was replaced with assay medium (Agilent Technologies) and cells were incubated at 37°C in non-CO_2_ incubator. Three measurements of oxygen consumption rate (OCR) and extracellular acidification rate (ECAR) were recorded pre- and postinjection of 1 µM oligomycin (Oligo), 0.5 µM FCCP, and 0.5 µM antimycin/rotenone (Ant/Rot) (Agilent Technologies) (14).

Using the Wave Desktop 2.3 software, seven parameters of mitochondrial respiration, basal OCR, ATP-linked OCR, OCR due to proton leak, maximal OCR, spare respiratory capacity, no-mitochondrial OCR, and ECAR, were calculated from the bioenergetic profiles obtained from the XF^e^24 extracellular flux analyzer, which has been outlined in detail previously (22). Briefly, basal OCR refers to the total baseline cellular respiration rate and includes respiration due to ATP production, proton leak (leak of protons across the inner mitochondrial membrane), and oxygen consumption due to nonmitochondrial processes (22). ATP-linked oxygen consumption is determined through the addition of the ATP synthase inhibitor oligomycin, which effectively shuts down ATP production due to oxidative phosphorylation. Any residual mitochondrial respiration/oxygen consumption noted at this point can then be attributed to proton leak (22). Maximal OCR is determined through the addition of the proton ionophore (uncoupler) FCCP, which increases inner mitochondrial membrane permeability to protons, increasing oxygen consumption and allowing for the assessment of the maximal oxygen consumption/respiration possible in the cells (22). Spare respiratory capacity is calculated through determining the difference between basal OCR and maximal OCR in the cells, and this reflects the amount of extra oxygen consumption/ATP production that can be achieved by the cells in response to increased energy demand (7). Nonmitochondrial respiration is the oxygen consumption due to nonmitochondrial processes. Although not well defined, this has been attributed to such processes as hydrogen peroxide production (3) and the enzymatic activity of oxygenases (4). Assessment of ECAR is primarily a measure of acid release and is related to lactic acid formation during glycolysis (14).

Basal respiratory capacity was recorded at the third readout of OCR just before oligomycin injection, whereas mitochondrial respiration due to proton leak was recorded at sixth OCR readout, which is just before FCCP injection. Maximal respiration was recorded as the highest OCR measurement following FCCP injection. ATP-linked respiration and spare respiratory capacity were calculated by subtracting OCR due to treatment with oligomycin from basal respiration and basal respiration from maximal respiratory capacity, respectively. Nonmitochondrial respiration was taken as the minimum OCR measurement after injection of Ant/Rot and was subtracted from all respiratory calculations. Values were normalized to total protein content. Two independent experiments were performed to assess mitochondrial respiration, each containing five biological replicates. Three measurements per time point were assessed.

#### RNA extraction and quantitative real-time PCR.

Isolation of total RNA from C2C12 myotubes was performed using TRIzol reagent, as per the manufacturer’s instructions (Invitrogen). Synthesis of cDNA was achieved using the iScript system (Bio-Rad Laboratories, Hercules, CA), according to the manufacturer’s protocol. Quantitative real-time PCR (qPCR) was undertaken using the SsoFast EvaGreen Supermix (Bio-Rad) and the CFX96 Real-Time PCR system (Bio-Rad). Transcript levels of target genes were normalized against the expression of the housekeeping gene *Gapdh*. Relative fold change in expression was calculated using the ΔΔcycle threshold (ΔΔC_T_) method. The sequences of the primers used in this manuscript are given in Table 1. All oligos pertaining to this study were purchased from Sigma-Aldrich (Singapore). All qPCR in this study was performed once with three biological replicates and two technical replicates per sample/treatment.

**Table 1.** Sequences of primers

Gene Symbol	Forward Primer Sequence	Reverse Primer Sequence
*Grk2*	AGAGGGACGTCAATCGGAGA	TTGCGGTACAGTTCCTGGTC
*mt-Co1*	GCACTGGTGGATGCCTTCT	TCTCTCGGGACTCCTTGATGA
*mt-Co2*	ACGTGCAACACCTGAGCGGT	GAAGGTGTCGGGCAGCAGGG
*mt-Co3*	CTACCAAGGCCACCACACTC	TCATGCTGCGGCTTCAAATC
*mt-Nd1*	TCCGAGCATCTTATCCACGC	GTATGGTGGTACTCCCGCTG
*mt-Nd4*	CCACTGCTAATTGCCCTCAT	CTTCAACATGGGCTTTTGGT
*Gapdh*	GATGATGACCCGTTTGGCTCC	ACGCTCGTGGAAAGAAAAGA

Table displays gene symbols and forward and reverse sequences of all primers used in the current study. *Grk2*, G protein-coupled receptor kinase 2; *mt-Co1*, cytochrome-*c* oxidase I; *mt-Co2*, cytochrome-*c* oxidase II; *mt-Co3*, cytochrome-*c* oxidase III; *mt-Nd1*, NADH dehydrogenase 1; *mt-Nd4*, NADH dehydrogenase 4.

#### Immunoblotting.

Proteins were isolated from myoblasts and myotubes using protein lysis buffer [50 mM Tris (pH 7.5), 250 mM NaCl, 5 mM EDTA, 0.1% NP-40, complete protease inhibitor cocktail (Roche, Indianapolis, MN), 2 mM NaF, 1 mM Na_3_VO_4_, and 1 mM phenylmethylsulfoxide (PMSF)]. Proteins were quantified using Bradford reagent (Bio-Rad). A total of 25 μg of each protein lysate was resolved on 4–12% BIS-TRIS precast gels (Invitrogen). Proteins were then transferred onto nitrocellulose membrane using either the Invitrogen iBlot 2 dry transfer system or the XCell II SureLock wet transfer system (Invitrogen). Membranes were then blocked overnight at 4°C in 5% milk in 1× Tris-buffered saline-Tween 20 (TBST), and proteins were hybridized with specific primary antibodies for 3 h in 5% milk/1× TBST. Membranes were then washed in 1× TBST, 5 times for 5 min each, before and after 1-h incubation with a 1:5,000 dilution of respective secondary antibodies, either goat anti-rabbit horseradish peroxidase (HRP) (cat. no. 1706515; Bio-Rad) or goat anti-mouse HRP (cat. no 1706516; Bio-Rad) antibodies. Antibody-bound proteins were detected using Western Lightning Chemiluminescence Reagent Plus (PerkinElmer, Boston, MA) and autoradiography films (Kodak). Protein levels were quantified and analyzed using the GS-800 calibrated densitometer (Bio-Rad) and analyzed using Quantity One imaging software (Bio-Rad). Details of the primary and secondary antibodies used in this study are provided in Table 2. The specificity of the anti-LC3B antibody has previously been demonstrated using a commercially available recombinant protein by Koukourakis et al. (31). The anti-mitofusin (MFN)1 antibody has been previously used to demonstrate increased levels of Mfn1 protein in liver tissue of high-fat diet-fed mice and in hepatocytes that display a swollen mitochondrial morphology (25). Previous target-specific siRNA knockdown studies have confirmed the specificity of the anti-P62 (76), anti-GRK2 (61), anti-PARKIN (36, 67), anti-MFN2 (79), anti-DRP1 (37), and anti-FIS1 (46) antibodies used in the current study. The number of experimental and biological replicates for immunoblot analysis is detailed in relevant figure legends.

**Table 2.** Details of antibodies

Antibody	Company	Catalog No.	Dilution	Blocking Solution
anti-GRK2	Santa Cruz	sc-562	1:1,000	5% milk
anti-GAPDH	Santa Cruz	sc-32233	1:1,000	5% milk
anti-PARKIN	Abcam	ab15954	1:1,000	5% milk
anti-MFN1	Abcam	ab126575	1:1,000	5% milk
anti-MFN2	Santa Cruz	sc-100560	1:1,000	5% milk
anti-FIS1	Santa Cruz	sc-98900	1:500	5% milk
anti-DRP1	Santa Cruz	sc-32898	1:5,000	5% milk
anti-P62	Abcam	ab91526	1:5,000	5% milk
anti-LC3B	Abcam	NB100-2220	1:500	5% milk
Goat anti-mouse HRP	Bio-Rad	1706516	1:5,000	5% milk
Goat anti-rabbit HRP	Bio-Rad	1706515	1:5,000	5% milk

Table displays the particulars of the antibodies used in the current study. Antibody name, source, catalog number, working dilution; and blocking solution are provided. GRK2, G protein-coupled receptor kinase 2; MFN1, mitofusin 1; MFN2, mitofusion 2; FIS1, mitochondrial fission 1 protein; DRP1, dynamin-related protein 1; HRP, horseradish peroxidase.

#### Mitotracker Red staining and assessment of mitochondria morphology using confocal microscopy.

Following 72 h doxycycline (2 µg/mL) induction, C2C12 myoblasts were seeded onto eight-well Permanox chambered slides at a density of 5,000 cells per well. After overnight attachment, myoblasts were treated with recombinant Mstn protein (3 µg/mL) for 24 h. To identify mitochondria, myoblasts were incubated for 30 min with 200 nM Mitotracker Red (CMX red Rosamine-based Mitotracker dye; Invitrogen). Cells were washed three times with PBS and subsequently fixed with paraformaldehyde (4%) in DMEM for 15 min. After fixation, cells were washed three times and were then mounted using SlowFade antifade reagent containing DAPI and analyzed using confocal microscopy (×60; Nikon).

To analyze mitochondrial morphology in control and GRK2-overexpressing C2C12 cells in the presence or absence of Mstn protein, the indexes of mitochondrial interconnectivity (area/perimeter ratio per mitochondrion), which is a measure of mitochondrial elongation, were quantified for each mitochondrion using the well-validated NIH ImageJ macros (Mitochondrial and Mitophagy Morphology macros; available at https://imagejdocu.tudor.lu/), as previously described (59), with minor modifications. The particular ImageJ macro was originally described by Dagda et al (6) and, importantly, has been used by several investigators since that time (5, 19, 66). Moreover, one recent study by Wiemerslage and LEE (74) further validated the Mitochondrial Morphology macro in dopamine neurons and analyzed the relationship and interdependency of individual parameters quantified by the macro (number of mitochondria, area, elongation, interconnectivity) under various conditions using principle component analysis. To quantify mitochondrial interconnectivity in C2C12 cell, between 10 and 15 high-resolution RGB images (TIFF, 1,020 × 1,020 pixels) were captured for each condition using confocal microscopy and were analyzed for mitochondrial morphology. To account for possible swelling of mitochondria, the area/perimeter were normalized for the minor axis of an ellipse that was “fitted” onto each mitochondrion analyzed by the macro. The interconnectivity ratio [(area/perimeter ratio)/minor axis] per cell were averaged for 50–100 mitochondria per cell and subsequently averaged for the entire population size for each experimental condition (25–30 cells). A low average interconnectivity ratio for a specific experimental condition (e.g., Mstn-treated cells relative to control untreated cells) is indicative of mitochondrial fragmentation (fission).

#### MitoTracker Green staining.

Following 72 h doxycycline induction, C2C12 myoblasts were seeded onto six-well plates at the density of 15,000 cells/cm^2^. The next day, cells were treated with 2 μg/mL recombinant Mstn protein or an equal volume of dialysis buffer (control) and incubated at 37°C, 5% CO_2_. After 24 h, myoblasts were stained with 150 nM MitoTracker Green FM (Thermo Fisher Scientific, Waltham, MA) for 20 min at 37°C. Cells were washed twice with PBS and harvested in conical tubes by centrifuging at 300 *g* for 1 min. Cell pellets were resuspended in PBS, and FACS analysis was performed to detect MitoTracker Green FM fluorescence intensity using the FACSCanto II flow cytometry system (BD Biosciences, Franklin Lakes, NJ). Fluorescent intensity of 10,000 events from 3 replicate wells per experimental group was detected using the FITC channel and is represented as mean fluorescent intensity. Two technical replicates were performed.

#### Statistical analysis.

Statistical analysis was performed using two-tail Student’s *t*-test and ANOVA, using the Bonferroni post hoc test. Data are expressed as means ± SE and results were considered significant at *P* < 0.05. A description of experiment replicates is provided in relevant figure legends.

## RESULTS

### 

#### Mstn promotes the loss of GRK2 protein via the ubiquitin proteasome pathway.

**Fig. 1.**
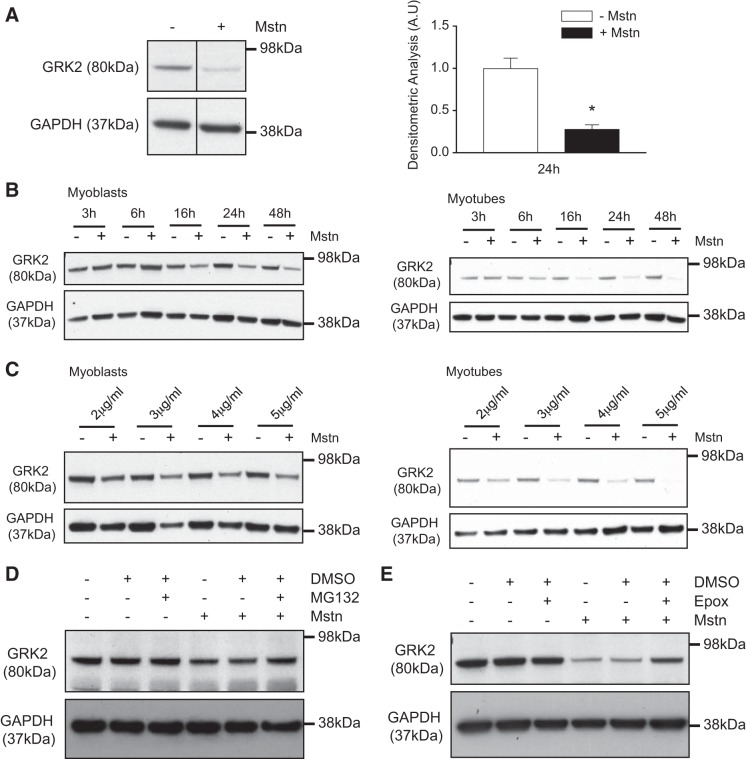
Excess myostatin (Mstn) leads to reduced G protein-coupled receptor kinase 2 (GRK2) protein levels. *A*, *left*: immunoblot (IB) analysis of GRK2 protein expression in C2C12 myotubes after 24 h treatment with (+) or without (−) Mstn. Relevant bands from the IB are shown. The levels of GAPDH were assessed as a loading control. *A*, *right:* densitometric analysis of protein levels (GRK2) normalized to GAPDH levels. Values represent means ± SE; 5 biological replicates from 3 independent experiments were performed and analyzed; *P < 0.05 (Student’s *t*-test). AU, arbitrary units. *B*: IB analysis of GRK2 protein content in both myoblasts (*left*) and myotubes (*right*) treated with (+) or without (−) Mstn over a time course (3, 6, 16, 24, and 48 h). The levels of GAPDH were assessed as a loading control. For each myoblast and myotube culture, one independent experiment with one biological replicate was performed. *C*: IB analysis of GRK2 protein levels in the absence (−) or presence (+) of increasing concentrations of Mstn protein (2, 3, 4, and 5 µg/mL) in both myoblasts (*left*) and myotubes (*right*). The levels of GAPDH were assessed as a loading control. For each myoblast and myotube culture, one independent experiment with one biological replicate was performed. *D*: IB analysis of GRK2 protein levels in myotubes treated with (+) or without (−) Mstn in the presence (+) or absence (−) of the proteasome inhibitor MG132 or vehicle control (DMSO). The levels of GAPDH were assessed as a loading control; *n* = 3 biological replicates from one independent experiment. *E*: IB analysis of GRK2 protein levels in myotubes treated with (+) or without (−) Mstn in the presence (+) or absence (−) of the proteasome inhibitor epoxomicin (Epox) or vehicle control (DMSO). The levels of GAPDH were assessed as a loading control. One biological replicate experiment and one independent experiment were performed.

Initially we investigated whether or not myostatin can modulate GRK2 expression. Immunoblot (IB) analysis revealed that treatment of C2C12 myotubes with recombinant myostatin protein (Mstn) resulted in a ~70% decrease in GRK2 protein content after 24-h treatment (Fig. 1*A*). We further noted both a time- and dose-dependent decrease in GRK2 protein levels in both C2C12 myoblasts and myotubes following treatment with Mstn (Fig. 1, *B* and *C*). However, Mstn-induced loss of GRK2 protein was more pronounced in C2C12 myotubes compared with Mstn-treated C2C12 myoblasts (Fig. 1, *B* and *C*).

Since Mstn has been shown to increase the activity of the UPP to promote loss of skeletal muscle proteins (44), we next evaluated whether or not Mstn promotes the loss of GRK2 protein through the UPP. As shown in Fig. 1, *D* and *E*, treatment of C2C12 myotubes with Mstn resulted in reduced protein levels of GRK2. However, treatment of C2C12 cells with two different specific proteasome inhibitors (MG132 and epoxomicin) was able to partially rescue the loss of GRK2 protein observed following Mstn treatment. (Fig. 1, *D* and *E*).

Taken together, these data suggest that Mstn is able to promote loss of GRK2 protein, through activation of the ubiquitin proteasome pathway (see summary in Fig. 4).

#### GRK2 and Mstn have differential effects on mitochondrial mass and OXPHOS gene expression in myotube cultures.

**Fig. 2.**
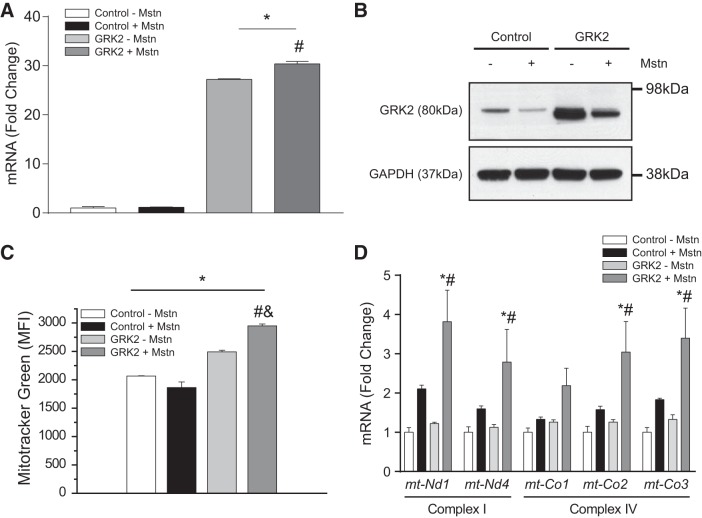
Overexpression of G protein-coupled receptor kinase 2 (GRK2) leads to increased mitochondrial content in muscle cells. *A*: quantitative (q)PCR analysis of *Grk2* expression in stable Control and GRK2-overexpressing C2C12 myotubes treated with (+) or without (−) myostatin (Mstn) for 24 h. Gene expression was normalized to the endogenous control *Gapdh*, using the ΔΔC_T_ method. Values represent means ± SE; *n* = 3 biological replicates from one independent experiment; *P < 0.05 vs. Control − Mstn; #*P* < 0.05 vs. GRK2 − Mstn. One-way ANOVA with Bonferroni correction was used for multiple comparisons. *B*: immunoblot (IB) analysis of GRK2 protein levels in Control and stable GRK2-overexpressing C2C12 myotubes treated with (+) or without (−) Mstn for 24 h. The levels of GAPDH were assessed as a loading control. Representative of at least 3 independent experiments. *C*: graph showing quantitative analysis of MitoTracker Green staining in Control and stable GRK2-overexpressing myotubes following treatment with (+) or without (−) Mstn. MFI. mean fluorescence intensity. Values represent means ± SE (2,065 ± 5 for Control − Mstn, 1,863 ± 100 for Control + Mstn, 2,491 ± 28 for GRK2 − Mstn, and 2,949 ± 34 for GRK2 + Mstn); *n* = 3 biological replicates from 1 independent experiment. *P < 0.05 vs. Control − Mstn; #*P* < 0.05 vs. GRK2 − Mstn; &*P* < 0.05 vs. Control + Mstn. One-way ANOVA with Bonferroni correction was used for multiple comparisons. *D*: qPCR analysis of mitochondrial encoded NADH dehydrogenase 1 (*mt-Nd1*), NADH dehydrogenase 4 (*mt-Nd4*), cytochrome-*c* oxidase I (*mt-Co1*), cytochrome-*c* oxidase II (*mt-Co2*), and cytochrome-*c* oxidase III (*mt-Co3*) expression in Control and stable GRK2-overexpressing C2C12 myotubes treated with (+) or without (−) Mstn for 24 h. Gene expression was normalized to the endogenous control *Gapdh*, using the ΔΔC_T_ method. Values represent means ± SE; *n* = 3 biological replicates from one independent experiment; *P < 0.05 vs. Control − Mstn; #*P* < 0.05 vs. GRK2 − Mstn. One-way ANOVA with Bonferroni correction was used for multiple comparisons.

It has been previously reported that GRK2 can target mitochondria in HEK293 cells to increase mitochondrial function and enhance ATP generation (18). On the other hand, myostatin is a myokine that promotes mitochondrial dysfunction and loss (39). Thus we next sought to determine *1*) the effect of GRK2 on mitochondrial mass and respiration in muscle cells, and *2*) whether or not GRK2 may play a role in Mstn regulation of mitochondria. To facilitate this, we generated doxycycline-inducible GRK2-overexpressing C2C12 cells, with GRK2 overexpression in myotubes subsequently confirmed through both qPCR (Fig. 2*A*) and immunoblot analysis (Fig. 2*B*). It is worth noting that despite significant overexpression of GRK2, Mstn treatment was still able to reduce GRK2 protein levels, but not *Grk2* mRNA expression, in GRK2-overexpressing C2C12 myotubes (Fig. 2, *A* and *B*). However, the levels of GRK2 protein remained elevated above endogenous levels compared with the control cell line, despite excess Mstn treatment (Fig. 2*B*).

Initially, we assessed mitochondrial mass through MitoTracker Green FM staining and subsequent FACS analysis. Results revealed a reduction in mitochondrial mass in response to Mstn treatment and an increase in mitochondrial mass upon overexpression of GRK2. (Fig. 2*C*). Conversely, a significant increase in mitochondrial mass was observed in GRK2-overexpressing myoblast cultures upon treatment with exogenous Mstn (Fig. 2*C*). Despite the increase in mitochondrial mass noted in control GRK2-overexpressing cells, the expression of critical OXPHOS genes (which encode for subunits of complex I and complex IV) was unaltered between untreated control and GRK2-overexpressing myotube cultures (Fig. 2*D*). Unexpectedly, the expression of the OXPHOS genes tended to increase in response to Mstn treatment (Fig. 2*D*), with the greatest increase in OXPHOS gene expression noted in Mstn-treated GRK2-overexpressing cells, when compared with Mstn-treated control cells (Fig. 2*D*).

#### GRK2 and Mstn influence mitochondrial fission and fusion in myotube cultures.

**Fig. 3.**
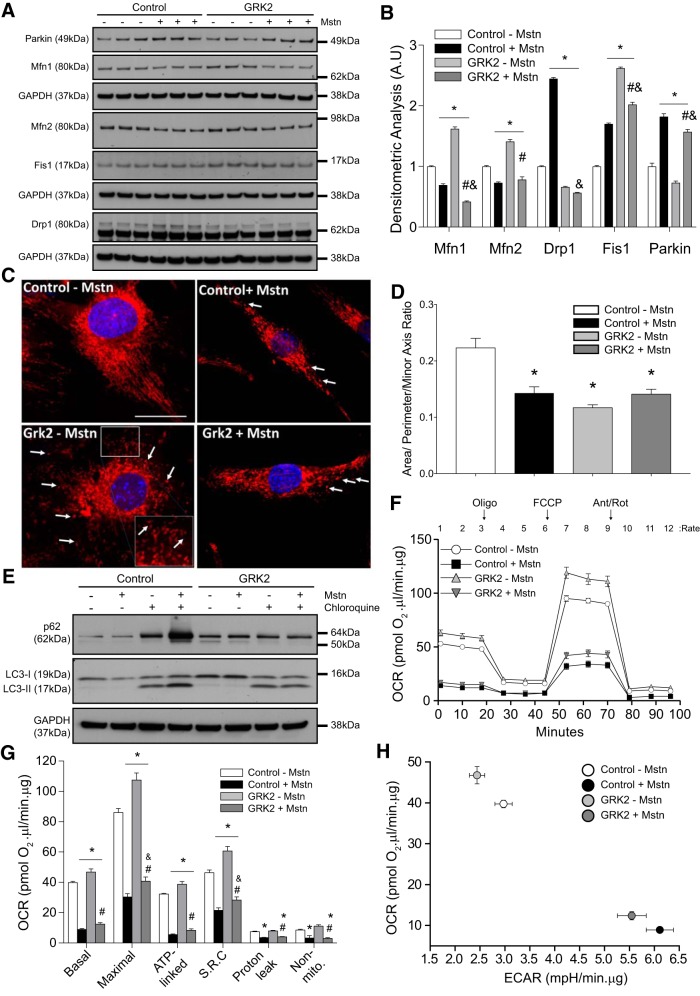
Overexpression of G protein-coupled receptor kinase 2 (GRK2) in myoblasts enhances mitochondrial respiration and reverses myostatin (Mstn)-induced autophagic flux. *A*: immunoblot (IB) analysis of mitofusin 1 and 2 (Mfn1/2), dynamin-related protein 1 (Drp1), mitochondrial fission 1 protein (Fis1), and Parkin protein levels in Control and stable GRK2-overexpressing C2C12 myotubes treated with (+) or without (−) Mstn for 24 h. The levels of GAPDH were assessed as a loading control; *n* = 3 biological replicates from one independent experiment. *B*: densitometric analysis of IB for Mfn1/2, Drp1, Fis1, and Parkin protein levels, normalized to GAPDH in Control and stable C2C12 myotubes-overexpressing GRK2 treated with (+) or without (−) Mstn for 24 h. Values represent means ± SE; *P < 0.05 vs. Control − Mstn; #*P* < 0.05 vs. GRK2 − Mstn; &*P* < 0.05 vs. Control + Mstn. One-way ANOVA with Bonferroni correction was used for multiple comparisons *B*: representative confocal micrographs of Control and GRK2-overexpressing myoblasts treated with (+) or without (−) Mstn and stained with MitoTracker Red to visualize mitochondria. Nuclei were counterstained with DAPI (blue). Scale bar = 40 µm. *Inset*: image was zoomed 40% from the original image and the white arrows are pointing to fragmented mitochondria (small and circular). *D*: quantitative imaged-based analyses of mitochondrial interconnectivity ratio [(area/perimeter)/minor axis] in paraformaldehyde-fixed control and GRK2-overexpressing C2C12 cells treated with (+) or without (−) recombinant Mstn protein. The bar graph shows data compiled from a representative experiment (data represent means ± SE; *n* = 20–35 biological replicates per condition from one independent experiment). One-way ANOVA with Bonferroni correction was used for multiple comparisons. *P < 0.05. *E*: IB analysis of p62, LC3-I, and LC3-II protein levels in Control and stable GRK2-overexpressing C2C12 myoblasts cotreated with (+) or without (−) Mstn for 12 h in the absence (−) or presence (+) of chloroquine. The levels of GAPDH were assessed as a loading control. Blots are representative of two independent experiments. *F*: graph showing the real-time oxygen consumption rate (OCR) in Control and stable GRK2-overexpressing C2C12 myotubes treated with (+) or without (−) Mstn, as assessed by the Seahorse XF^e^24 extracellular flux analyzer. Time points where oligomycin (Oligo), FCCP, and antimycin/rotenone (Ant/Rot) were injected (arrows) and the rate number where each OCR was measured are indicated. Values represent means ± SE of three independent measurements from five biological replicates. *G*: graph showing quantification of basal, maximal, ATP-linked, and nonmitochondrial (Non-mito) respiration, spare respiratory capacity (SRC), and respiration due to proton leak in Control and stable GRK2-overexpressing C2C12 myotubes treated with (+) or without (−) Mstn. All OCR values were normalized to total protein. Values represent means ± SE of three independent measurements from five biological replicates. Two different experiments were performed. *P < 0.05 vs. Control − Mstn; #*P* < 0.05 vs. GRK2 − Mstn; &*P* < 0.05 vs. Control + Mstn. One-way ANOVA with Bonferroni correction was used for multiple comparisons. *H*: graph showing OCR vs. extracellular acidification rate (ECAR) in Control and stable GRK2-overexpressing C2C12 myotubes treated with (+) or without (−) Mstn. Values represent mean ± SE of three independent measurements from five biological replicates. Two different experiments were performed.

Next, we investigated the role of GRK2 and Mstn in mitochondrial structure/dynamics through analysis of mitochondrial fission and fusion markers. Western blot analysis revealed a significant reduction in the protein levels of mitochondrial fusion markers, MFN1 and MFN2, upon Mstn treatment, in both control and GRK2-overexpressing C2C12 myotubes (Fig. 3, *A* and *B*). Furthermore, a significant increase in the levels of the mitochondrial fission marker proteins Drp1 and Fis1 and the mitochondrial E3 ligase PARKIN was observed upon Mstn treatment of control cells, with significantly increased levels of both Fis1 and PARKIN also noted in Mstn-treated GRK2-overexpressing C2C12 myotubes (Fig. 3, *A* and *B*). These data suggest that Mstn treatment is associated with reduced mitochondrial fusion and increased mitochondrial fission in C2C12 myotube cultures. A significant increase in the levels of Fis1 was also noted in untreated GRK2-overexpressing C2C12 myotubes, when compared with untreated control cells; however, in contrast to what was observed following Mstn treatment, the levels of Drp1 and Parkin remained low and were in fact slightly reduced in untreated GRK2-overexpressing C2C12 myotubes, when compared with untreated controls (Fig. 3, *A* and *B*). Interestingly, significantly increased levels of both MFN1 and MFN2 were observed in untreated GRK2-overexpressing C2C12 myotubes, when compared with controls (Fig. 3, *A* and *B*).

We next stained control and GRK2-overexpressing myoblasts, treated with or without Mstn protein, with Mitotracker Red to visualize mitochondria and to assess for qualitative changes in mitochondria morphology (Fig. 3*C*). Through using semiautomated macros that determine the mitochondrial interconnectivity ratio (area/perimeter normalized to the minor axis of an ellipse) (59), we observed that untreated cells contained interconnected mitochondria, as evident by long tubular mitochondrial networks (Fig. 3, *C* and *D*). However, Mstn treatment of cells led to a robust fragmentation of mitochondrial networks and a decreased mitochondrial interconnectivity ratio (Fig. 3*D*). Paradoxically, inducible expression of GRK2 also resulted in a decreased mitochondrial interconnectivity ratio per cell (Fig. 3*D*). The combination of GRK2 overexpression and Mstn treatment resulted in a partial reversal of mitochondrial fragmentation induced by GRK2 alone, back to levels similar to cells treated with Mstn alone. However, the overall levels of fragmentation were still significantly lower when compared with untreated control cells (Fig. 3*D*). Based on the image analysis, our data show that both GRK2 treatment and Mstn treatment induce mitochondrial fragmentation/fission, although the molecular mechanism through which both proteins promote reduced mitochondrial interconnectivity is distinct, as per our Western blot data (altered MFN1/2 levels and increased Drp1 versus Fis 1 levels; Fig. 3*B*).

#### GRK2 overexpression prevents the increased autophagic flux observed in response to Mstn treatment.

It is well established that excess myostatin leads to increased autophagy (35, 73). Mitophagy is the selective process by which damaged/defective mitochondria are targeted for lysosomal-mediated degradation (30). Once different outer mitochondrial membrane-localized proteins are ubiquitinated, by E3 ligases including Parkin, mitochondria are “flagged” and targeted for degradation by the ubiquitin-binding adaptor protein P62/SQSTM1, which in turn associates with LC3 in the autophagosome, leading to the engulfment and degradation of mitochondria (51). During this process, the LC3 isoform I is conjugated to phosphatidylethanolamine to form a membrane-bound form of LC3, termed LC3-II, which remains bound to autophagosome until it is targeted for degradation by the lysosome (68). To determine whether or not GRK2 plays a role in autophagy, we next assessed autophagic flux in dialysis buffer and Mstn-treated control and GRK2-overexpressing cells, in the presence or absence of chloroquine, a lysosomotropic agent that inhibits autophagy (64). As observed in Fig. 3*E*, chloroquine treatment resulted in the accumulation of both p62 and LC3-II in control cells and of LC3-II in GRK2-overexpressing cells, consistent with a blockade in autophagy (Fig. 3*E*). However, upon Mstn treatment, we observed a noticeable increase in p62 and LC3-II accumulation in chloroquine-treated control cells, when compared with dialysis buffer-treated controls (Fig. 3*E*), indicating increased autophagic flux in response to Mstn treatment. Interestingly, no difference in p62 or LC3-II accumulation was observed in Mstn-treated GRK2-overexpressing cells in the presence of chloroquine, when compared with control cells treated with chloroquine. These data suggest that overexpression of GRK2 prevents the overt autophagic flux induced upon Mstn treatment in myotube cultures, which is consistent with the increased mitochondrial mass noted in Mstn-treated GRK2-overexpressing myoblasts (Fig. 2*C*).

#### Mstn treatment impairs, while GRK2 overexpression increases, mitochondrial respiratory capacity in C2C12 myoblasts.

**Fig. 4.**
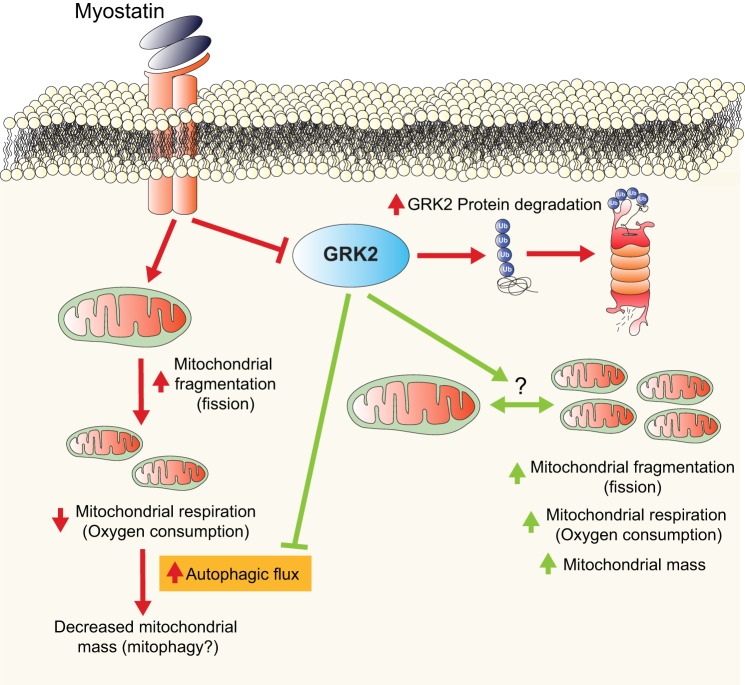
G protein-coupled receptor kinase 2 (GRK2) regulates mitochondrial respiratory function and impairs myostatin (Mstn)-mediated autophagy in muscle cells. Mstn signaling (red lines and arrows) in muscle cells results in loss of GRK2 protein through a mechanism involving the ubiquitin-proteasome pathway. Furthermore, Mstn treatment leads to increased mitochondrial fragmentation (consistent with mitochondrial fission), impaired mitochondrial respiration and decreased mitochondrial mass, which was associated with increased autophagic flux in muscle cells. Taken together, we surmise that Mstn may act to stimulate autophagy-mediated clearance of mitochondria, or mitophagy, in muscle cells. Overexpression of GRK2 (green lines and arrows), although not able to overcome Mstn-induced impairment of mitochondrial respiration, blocked the increased autophagic flux promoted by Mstn. Moreover, elevated GRK2 levels resulted in mitochondrial fragmentation, which was associated with an increase in both mitochondrial mass and mitochondrial respiration. Given that overexpression of GRK2 resulted in mitochondrial fragmentation and altered the levels of critical mitochondrial fusions/fission proteins we speculate a role for GRK2 in regulating the balance between mitochondria fusion and fission in muscle cells (indicated by the?). Arrows represent stimulation and blunt-ended lines represent inhibition.

We next evaluated mitochondrial respiration in control and GRK2-overexpressing myotubes by measuring OCR and ECAR by employing the XF^e^24 Extracellular Flux Analyzer (Agilent Technologies). In this system, OCR is used to measure real-time mitochondrial respiration and ECAR is used to measure glycolysis (14). Extracellular flux analysis revealed a significant reduction in overall OCR in myotubes treated with Mstn (Fig. 3, *F* and *G*). Subsequent quantification of real-time OCR data revealed a significant reduction in basal OCR, ATP-linked OCR, which reflects ATP production through oxidative phosphorylation, maximal OCR (maximal respiration possible in the cells), and spare respiratory capacity (amount of extra ATP production that can be achieved by the cells in response to increased energy demand) following Mstn treatment (Fig. 3*G*). In addition, Mstn treatment resulted in a significant reduction in the OCR due to proton leak (leak of protons across the inner mitochondrial membrane) as well as nonmitochondrial respiration, which is OCR due to nonmitochondrial processes in the cells (Fig. 3*G*). Interestingly, GRK2 overexpression led to a significant increase in the OCR of C2C12 myotubes (Fig. 3, *F* and *G*), with a significant increase in basal OCR, maximal OCR, ATP-linked OCR, and spare respiratory capacity noted upon overexpression of GRK2 (Fig. 3*G*), suggesting that elevated GRK2 has a positive effect on cellular respiration, enhancing maximal cell respiration and the potential to produce extra ATP in times of increased energy demand (spare respiratory capacity). Importantly, we noted a statistically significant, albeit only a very modest, reversal of Mstn-mediated repression of maximal OCR and spare respiratory capacity upon overexpression of GRK2 (Fig. 3*G*). The graph shown in Fig. 3*H* is a visual representation of the metabolic phenotype in cells and reveals that untreated GRK2-overexpressing cells are more aerobic, when compared with untreated control cells (Fig. 3*H*). In addition, analysis revealed that Mstn treatment resulted in a robust increase in glycolysis and decreased aerobic respiration in myotube cultures, as evident by the increase in ECAR and concomitant reduction in OCR, respectively. Taken together these data suggest that while overexpression of GRK2 has a positive effect on mitochondrial respiration and can block Mstn-mediated autophagy in muscle cells, overexpression of GRK2 is not able to completely reverse the detrimental effect of Mstn on mitochondrial respiration (see summary in Fig. 4).

## DISCUSSION

In this report, we have undertaken studies to explore GRK2 function in muscle cells and the role that GRK2 plays in myostatin-mediated regulation of mitochondrial respiration. Herein, we have shown that overexpression of GRK2 in muscle cells leads to increased mitochondrial mass and respiration, as measured through oxygen consumption rate. In addition, our data suggest that GRK2 modulates mitochondrial dynamics, as inducible overexpression of GRK2 altered the levels of key regulators of mitochondrial fission and fusion and ultimately resulted in increased mitochondrial fragmentation. Excess Mstn also altered the levels of mitochondrial fusion and fission markers and further led to increased mitochondrial fragmentation; however, in contrast to what was observed in GRK2-overexpressing myoblasts, excess Mstn resulted in reduced mitochondrial mass, increased autophagic flux, and impaired mitochondrial respiration in muscle cells. Importantly, although elevated GRK2 levels was able to prevent the Mstn-mediated increase in autophagic flux, overexpression of GRK2 was unable to rescue the impaired mitochondrial respiration noted upon Mstn treatment. Our findings support a beneficial role for GRK2 in increasing mitochondrial respiration and preventing overt autophagy and loss of mitochondrial mass in skeletal muscle cells.

As we have observed that Mstn represses the protein levels of GRK2 (Figs. 1 and 2) but has no significant inhibitory effect on *Grk2* mRNA expression (Fig. 2*A*), we propose that Mstn regulates GRK2 levels posttranscriptionally. This is quite consistent with the involvement of the ubiquitin-proteasome pathway (UPP) in Mstn-induced repression of GRK2 protein levels that we have described (Fig. 1, *D* and *E*). Given this, we propose that GRK2 protein may be targeted for degradation through the UPP in response to Mstn treatment (Fig. 4). Mstn has been shown to upregulate both atrogin-1 and MuRF1 E3 ligases to promote UPP-mediated protein degradation in conditions of muscle wasting (40, 44); thus we speculate that myostatin may signal through the E3 ligases atrogin-1 and/or MuRF1 to target and degrade GRK2 protein. Moreover, Salcedo et al. (60) have revealed that in HeLa and HEK293 cells GRK2 is targeted by the E3-ubiquitin ligase Mdm2 for degradation through the UPP upon β_2_-adrenergic receptor stimulation. Given that Mdm2 is expressed in muscle cells (17), it is quite possible that myostatin may signal through Mdm2 to regulate GRK2. However, future studies will need to be performed to further clarify the specific molecular mechanism(s) through which Mstn targets and represses GRK2 protein levels in muscle cells.

It is important to highlight that we noted a more pronounced repression of GRK2 protein levels in Mstn-treated myotube cultures, when compared with myoblast cultures (Fig. 1, *B* and *C*). Although the exact reason for this phenomenon remains to be defined, it is noteworthy to mention that the levels of the canonical myostatin signaling target Smad3 (24) are increased during myogenic differentiation (9, 78). Thus we speculate that the greater inhibitory effect of myostatin on GRK2 may be due to increased availability of Smad3 and subsequent downstream signaling in myotube cultures. However, future studies will need to be undertaken to confirm this.

Fusco et al. (18) have recently shown that GRK2 overexpression in HEK293 cells led to increased ATP production and mitochondrial biogenesis and that loss of GRK2 from skeletal muscle in vivo reduces ATP production. In agreement with this, we find increased mitochondrial mass, OCRs, and cellular respiration, which are consistent with enhanced mitochondrial respiratory function, in GRK2-overexpressing skeletal muscle cells. Increased oxygen consumption was also associated with reduced ECAR in GRK2-overexpressing myoblasts. Similar results have been observed previously in myoblast cultures (10) and suggest that these cells rely on oxidative phosphorylation, as opposed to glycolysis, to meet cellular energy demands. In contrast, upon Mstn addition to control cells, we observed significantly decreased oxygen consumption in muscle cells (Fig. 3*F*). In addition to reduced basal mitochondrial respiration, Mstn treatment led to significantly reduced maximal mitochondrial respiration when compared with controls. This could indicate diminished availability of substrate (although comparable medium constituents are maintained across all cell cultures), disruption of the electron transport chain, or reduced mitochondrial mass (22). In agreement with this, reduced mitochondrial mass was seen in response to Mstn treatment of C2C12 cells (Fig. 2*C*). Together with reduced maximal respiration, we also noted reduced spare respiratory capacity upon Mstn treatment, which suggests that Mstn-treated cells may have reduced ability to respond to increased energy demand, when compared with control cells. We further observed reduced ATP-linked respiration upon Mstn treatment, which could indicate a reduced requirement for ATP, reduced availability of substrate or importantly, impaired function of the electron transport chain, and subsequent oxidative phosphorylation (22). Moreover, reduced OCR and a concomitant increase in ECAR was noted in Mstn-treated cells, consistent with a switch from predominantly aerobic respiration to glycolysis in these cells. Taken together, these observations are consistent with previously published work, revealing that excess Mstn leads to mitochondrial dysfunction and reduced oxygen consumption (39). Unexpectedly, despite increased mitochondrial mass we did not find a rescue of Mstn-mediated impairment of oxygen consumption and cellular respiration upon overexpression of GRK2, although a very minor rescue of maximal respiration and related spare respiratory capacity was noted. Taken together, these data suggest that overexpression of GRK2 is not able to compensate for the deleterious effect of Mstn on mitochondrial respiration and, due to the increased mitochondrial mass noted, conceivably leads to an accumulation of dysfunctional mitochondrial in these cells.

Increased expression of OXPHOS genes (subunits of complex I and IV) was noted in both Mstn-treated control cells (albeit not statistically significant) and Mstn-treated GRK2-overexpressing cells. A similar increase in complex IV OXPHOS gene expression has been observed in fibroblasts derived from patients with ATP synthase deficiency, independently of changes in mtDNA (21). Moreover, increased mRNA expression of OXPHOS genes has been noted in diseases associated with additional mitochondrial complex deficiencies (57). Therefore, we speculate that the increased mRNA expression of OXPHOS genes observed in Mstn-treated cells may act to compensate for the reduced oxygen consumption/mitochondrial respiration noted in response to Mstn treatment.

It is noteworthy to mention that a more pronounced increase in OXPHOS gene expression was observed in GRK2-overexpressing cells following Mstn treatment. Previous work by Sorriento et al. (65) have revealed that macrophages treated with LPS exhibit enhanced translocation and accumulation of GRK2 in mitochondria, which in turn was associated with elevated expression of cytochrome *b* and NADHd (complex III and I, respectively). This may help to explain the OXPHOS gene expression pattern noted in Mstn-treated GRK2-overexpressing myoblasts. However, further studies will need to be undertaken to confirm this hypothesis.

In eukaryotic cells, mitochondrial content is tightly controlled through pathways that modulate mitochondrial biogenesis and mitochondrial clearance (autophagy/mitophagy) (8). Mstn robustly increases autophagic flux in myoblasts (Fig. 3*E*). Moreover, we further find that Mstn treatment leads to mitochondrial fragmentation (Fig. 3*D*), impaired mitochondrial respiration (Fig. 3, *F* and *G*), and a reduction in mitochondrial mass (Fig. 2*C*). Taken together these data suggest that Mstn treatment disrupts mitochondrial respiration and leads to decreased mitochondrial mass in muscle cells. It is important to mention that while the increased autophagic flux and decreased mitochondrial mass are consistent with enhanced autophagy-mediated mitochondria clearance or mitophagy, a more direct measure of mitophagy would need to be performed to confirm this. Interestingly, GRK2-overexpressing cells, when treated with Mstn, exhibited decreased autophagic flux, which was supported by reduced chloroquine-mediated accumulation of LC3-II and p62 in response to Mstn treatment, when compared with controls. These data suggest that overexpression of GRK2 blocks the overt autophagic flux induced by Mstn treatment, which would most certainly account for the increased mitochondrial content observed in Mstn-treated GRK2-overexpressing myoblasts (Fig. 2*C*). Furthermore, given that GRK2 reduces autophagic flux in myoblasts and that Mstn treatment leads to a reduction in GRK2 protein levels in muscle cells, it is interesting to surmise that Mstn may repress GRK2 protein to facilitate autophagy-mediated clearance of mitochondria. However, further work will need to be undertaken to validate this mechanism in muscle cells.

The processes of mitochondrial fusion and fission are tightly regulated and are critically involved in governing mitochondria turnover, as evidenced by previous work (71). Here, we show that GRK2-overexpressing cells exhibited increased levels of both mitochondrial fusion (Mfn1/2) and fission (Fis1) proteins, suggesting that overexpression of GRK2 promotes increased mitochondrial fission/fusion, which is consistent with recent work assessing GRK2 function during ionizing radiation-induced mitochondrial damage (16). In contrast to this, Mstn treatment tended to decrease the levels of both Mfn1 and Mfn2 in both control and GRK2-overexpressing myoblasts, suggesting that Mstn treatment may impair mitochondrial fusion. In addition, Mstn treatment led to elevated levels of the mitochondrial fission markers Drp1 and Fis1. However, it is interesting to note that while elevated Fis1 levels were maintained in Mstn-treated GRK2-overexpressing myoblasts the Mstn-mediated increase in Drp1 was ablated in GRK2-overexpressing cells, revealing that GRK2 may have an inhibitory role in controlling Drp1 levels. Furthermore, given that Mstn treatment leads to elevated Drp1 and Fis1 (Fig. 3, *A* and *B*) and that overexpression of GRK2 increases the expression of Fis1 but not Drp1 (Fig. 3, *A* and *B*), we propose that the changes in mitochondrial dynamics observed in response to either Mstn treatment or GRK2 overexpression may occur through distinct mechanisms. Consistent with this, recent studies have revealed that Fis1 can regulate mitochondrial morphology independently of Drp1 (50).

It is important to mention that despite differential regulation of fusion and fission proteins by Mstn and GRK2, a similar reduction in mitochondrial interconnectivity, consistent with increased mitochondrial fragmentation, was noted between GRK2-overexpressing myoblasts and Mstn-treated control and GRK2-overexpressing cells, when compared with untreated controls (Fig. 3*D*). Importantly, despite a similar level of mitochondrial fragmentation, overexpression of GRK2 alone led to increased mitochondrial respiration. We propose that the differences in mitochondrial respiration observed may be linked to maintenance of mitochondrial membrane potential in GRK2-overexpressing cells. Most certainly, previous work has revealed that fragmentation of mitochondria does not necessarily lead to reduced membrane potential (47) and more importantly, overexpression of GRK2 has been linked with maintenance of mitochondrial membrane potential in HEK293 cells in response to ionizing radiation-induced damage (16). Moreover, we find that the levels of the E3 ligase Parkin, which is recruited to damaged/defective mitochondria with low membrane potential to mediate their removal by autophagosomes (48), remained unchanged in control GRK2-overexpressing cells.

In conclusion, here we have described a beneficial role for GRK2 in regulating mitochondrial respiratory function and further reveal that excess GRK2 is able to influence autophagic flux in skeletal muscle cells (Fig. 4). Although GRK2 has previously been shown to have a protective role in response to acute mitochondrial damage (16), we find that GRK2 is unable to prevent the significant deleterious effects of Mstn treatment on mitochondrial respiration in muscle cells (Fig. 4).

## GRANTS

This study was funded by the Agency for Science, Technology, and Research (A*STAR), Singapore and partially funded by NIH Grants GM103554 and NS105783-01 (to R. K. Dagda). We are also indebted to Coordenação de Aperfeiçoamento de Pessoal de Nível Superior (5662-13-3), Conselho Nacional de Desenvolvimento Científico e Tecnológico, and Fundação de Amparo à Pesquisa do Estado de São Paulo, Brazil, for financial support.

## DISCLOSURES

No conflicts of interest, financial or otherwise, are declared by the authors.

## AUTHOR CONTRIBUTIONS

L.H.M. and C.M. conceived and designed research; L.H.M., J.A., N.P., and R.K.D. performed experiments; L.H.M., J.A., N.P., R.K.D., and C.M. analyzed data; L.H.M., J.A., N.P., R.K.D., and C.M. interpreted results of experiments; L.H.M. prepared figures; L.H.M. and C.M. drafted manuscript; L.H.M., J.A., N.P., R.K.D., and C.M. edited and revised manuscript; L.H.M., J.A., N.P., R.K.D., and C.M. approved final version of manuscript.
